# Characterization of CD56^+^ Dendritic-Like Cells: A Normal Counterpart of Blastic Plasmacytoid Dendritic Cell Neoplasm?

**DOI:** 10.1371/journal.pone.0081722

**Published:** 2013-11-29

**Authors:** Yohei Osaki, Akihiko Yokohama, Akio Saito, Kenichi Tahara, Kunio Yanagisawa, Yoshiyuki Ogawa, Takuma Ishizaki, Takeki Mitsui, Hiromi Koiso, Makiko Takizawa, Hideki Uchiumi, Takayuki Saitoh, Hiroshi Handa, Hirokazu Murakami, Norifumi Tsukamoto, Yoshihisa Nojima

**Affiliations:** 1 Department of Medicine and Clinical Science, Gunma University Graduate School of Medicine, Maebashi, Gunma, Japan; 2 Division of Blood Transfusion Service, Faculty of Medicine, Gunma University Hospital, Maebashi, Gunma, Japan; 3 Oncology Center, Gunma University Hospital, Maebashi, Gunma, Japan; 4 Gunma University School of Health Sciences, Faculty of Medicine, Gunma University, Maebashi, Gunma, Japan; Istituto Superiore di Sanità, Italy

## Abstract

Blastic plasmacytoid dendritic cell neoplasm (BPDCN) is a rare hematological malignancy. Plasmacytoid DCs (pDCs), which are defined as lineage marker (Lin)^−^HLA−DR^+^CD56^−^CD123^+^CD11c^−^ cells, are considered to be the normal counterpart of BPDCNs. However, BPDCN can be distinguished from pDCs by uniform expression of CD56. In this study, to identify a normal counterpart of BPDCN, we searched for a Lin^−^HLA−DR^+^CD56^+^ population and focused on a minor subpopulation of Lin^−^DR^+^CD56^+^CD123^+^CD11c^−^ cells that we designated as pDC-like cells (pDLCs). pDLC constituted 0.03% of peripheral blood mononuclear cells (PBMCs), and the pDLC/pDC ratio was higher in bone marrow cells than in PBMCs. pDLC clearly expressed BDCA2, BDCA4, and myeloid antigens, which are frequently expressed by BPDCN. pDLCs exhibited modest expression of Toll-like receptors and produced less interferon-α after CpG stimulation, but presented very low endocytic ability unlike mDCs. These functional differences were attributed to the expression profile of transcriptional factors. After *in vitro* culture with Flt3-ligand and GM-CSF, pDLCs expressed CD11c and BDCA1. These data suggested that pDLCs are a distinct subpopulation, with an immunophenotype similar to BPDCNs. Moreover, our results indicate that pDLCs might be immature DCs and might contribute to the immunophenotypical diversity of BPDCNs.

## Introduction

Blastic plasmacytoid dendritic cell neoplasm (BPDCN) is a rare type of myeloid neoplasm. Its diagnosis is based on its CD4^+^CD56^+^CD123^+^HLA−DR^+^ cell-surface marker profile and the absence of lineage marker (Lin) expression[1,2]. This neoplasm often involves the skin and bone marrow, and its clinical course is usually aggressive[3]. Although many studies have described BPDCN, descriptions of its phenotype and function, which are critical for accurate diagnosis, vary between studies[4,5]; thus, the features of BPDCN might be heterogeneous.

A normal counterpart of BPDCN is thought to be the plasmacytoid dendritic cell (pDC). pDCs are a subset of the heterogeneous dendritic cell (DC) family that serve a critical role in the immune system as pathogen sensors and activators of adaptive immunity[6]. Human peripheral blood DCs are phenotypically defined as leukocytes that lack markers of other leukocyte lineages, e.g., CD3 (T cell), CD14/16 (monocyte), CD19 (B cell), or CD56 (natural killer (NK) cell), and express high levels of major histocompatibility complex (MHC) class II molecules[6,7]. CD11c and CD123 expression can be used to categorize Lin^−^HLA−DR^+^ blood DCs into CD123^+^CD11c^−^pDCs and CD123^−^CD11c^+^ myeloid dendritic cells (mDCs)[8]. In addition, pDCs are distinguishable by the expression of CD303 (BDCA2) and CD304 (BDCA4), whereas mDCs express BDCA1 and BDCA3[9]. pDCs produce interferon (IFN)-α and induce Th2 polarization of naïve CD4^+^ T cells in response to various stimuli[1]; both these functions are known to affect various disease outcomes[10,11]. Although DCs are thought to be derived from bone marrow, the pathways that control DC differentiation and molecular regulation are not completely understood. Furthermore, the common dendritic progenitor (CDP), which can directly differentiate into a pDC or mDC, has been identified in mice[12-14]. In addition, the neural cell adhesion molecule (NCAM), which corresponds to CD56 in humans, is known to mark immature DCs[15].

Although pDCs are a normal counterpart of BPDCN, BPDCNs differ from pDCs in several ways; for example, BPDCN expresses CD56, CD13, and CD33, and occasionally develops concomitantly with acute myeloid leukemia[3,16]. These observations suggest that the oncogenic origin of BPDCN is not equivalent to that of pDCs in the blood. Moreover, several studies have reported that a small population of CD56^+^pDCs in the blood are immunophenotypically similar to BPDCN[17,18]. Although the immunophenotype, function, and differentiation of pDCs have been extensively investigated, this minor population of CD56^+^pDCs has not been previously characterized.

In this study, we focused on the Lin^−^HLA−DR^+^CD56^+^ population in steady-state condition to identify a normal counterpart of BPDCN. In order to understand the relationship between BPDCN and its normal counterpart as well as its oncogenic origin, we investigated the characteristics, immunophenotype, function, and transcription factor expression patterns of CD56^+^pDC, pDC, and mDC.

## Materials and Methods

### Cell isolation

This study was approved by the local institutional review board of Gunma University (Gunma, Japan). Peripheral blood mononuclear cells (PBMCs) were isolated from healthy donors by Ficoll centrifugation after obtaining written informed consent from the respective patients. Lineage markers were defined as CD3, CD14, CD16, and CD19. Isolated PBMCs were stained with biotinylated anti-human CD3 (BD Pharmingen, San Diego, CA, USA), CD14 (BioLegend, San Diego, CA, USA), CD16 (BioLegend), and CD19 (BioLegend) antibodies. Subsequently, biotinylated Lin+ cells and red blood cells (RBCs) were depleted using anti-biotin microbeads (Miltenyi Biotec, Bergisch Gladbach, Germany) and CD235a microbeads (Miltenyi Biotec), respectively. These cells were analyzed by flow cytometry, if not otherwise specified. For cell sorting, lineage-depleted, DC-enriched PBMC were further purified by positive selection with HLA-DR microbeads (Miltenyi Biotec). Purified HLA−DR^+^ cells were labeled with allophycocyanin–Cy7 (APC^−^Cy7, BD Pharmingen)-conjugated anti-streptavidin (BioLegend), peridinin–chlorophyll protein (PerCP)-conjugated anti-human HLA-DR (BD Pharmingen), phycoerythrin cyanine 7 (PE-Cy7)-conjugated anti-human CD56 (Becton Dickinson, San Jose, CA, USA), APC-conjugated anti-human CD123 (BD Pharmingen), or fluorescein isothiocyanate (FITC)-conjugated anti-human CD11c (Miltenyi Biotec). Subsequently, the target population was isolated by fluorescence-activated cell sorting (FACS) using a FACS Aria II cell sorter (Becton Dickinson, San Jose, CA, USA). Purity of the sorted cells was always >95%.

### Cell culture

Sorted cells were cultured in 96-well round-bottom plates in RPMI1640 medium supplemented with 10% fetal bovine serum and containing 100 ng/mL recombinant human Flt3-ligand (Flt3-L; R&D Systems, Minneapolis, MN, USA) and 100 ng/mL granulocyte colony-stimulating factor (GM-CSF; R&D Systems) for six days. The medium was changed every three days.

### Flow cytometry

In order to identify and quantify cell populations, PBMCs were labeled with FITC-conjugated anti-human CD3 (BD Pharmingen), FITC-conjugated anti-human CD14 (BD Pharmingen), FITC-conjugated anti-human CD16 (BD Pharmingen), FITC-conjugated anti-human CD19 (BD Pharmingen), PerCP-conjugated anti-human HLA-DR (BD Pharmingen), PE-Cy7-conjugated anti-human CD56 (Becton Dickinson & Co.), APC-conjugated anti-human CD123 (BD Pharmingen), and PE-conjugated anti-human CD11c (BD Pharmingen). After negative selection, enriched PBMCs were labeled with APC-Cy7-conjugated streptavidin (BioLegend), PerCP-conjugated anti-human HLA-DR (BD Pharmingen), APC-conjugated anti-human CD123 (BD Pharmingen), and FITC-conjugated anti-human CD11c (Miltenyi Biotec GmbH) for subsequent analysis. Furthermore, in some experiments, enriched PBMCs were labeled with PE-conjugated anti-human antibodies. Intracellular staining was performed according to the instructions provided by the manufacturer. An FACSCanto II flow cytometer (BD Bioscience) was used for all flow cytometric analyses, and data were analyzed with FlowJo software, version 9.5.2 (Treestar, Inc., San Carlos, CA, USA).

### RNA isolation, cDNA preparation, and quantitative real-time polymerase chain reaction (qRT-PCR)

RNA was isolated using the RNeasy Micro Kit (Qiagen, Hilden, Germany) and was transcribed to cDNA using the High Capacity RNA-to-cDNA Kit (Applied Biosystems, Carlsbad, CA, USA) as per the instructions provided by the manufacturer. qRT-PCR was performed using the 7300 Real-Time PCR System (Applied Biosystems). Taqman Gene Expression Assays (Applied Biosystems) were as follows: Toll-like receptor (TLR) 2 Hs01872448_s1, TLR4 Hs00152939_m1, TLR7 Hs01933259_s1, TLR9 Hs00370913_s1, E2-2 Hs00162613_m1, ID2 Hs04187239_m1, IRF8 Hs00175238_m1, PU.1 Hs00745162_s1, SPIB Hs00162150_m1, IFN-α Hs00855471_g1, IL12 Hs 01073447_m1, IL10 Hs00961622_m1, IL6 Hs00985639_m1, TNF-α Hs01113624_g1, and GAPDH Hs02758991_g1. GAPDH was used as an internal control. Thereafter, we quantified the results for each gene of each population and compared them with average PBMC values from healthy donors.

### Cytokine production assay

Sorted cells were cultured with 1 μM CpG oligodeoxynucleotide 2216 (CpG; Enzo Life Sciences, Farmingdale, NY, USA) or 100 ng/mL lipopolysaccharide (LPS; Sigma-Aldrich, St. Louis, MO, USA). The cells were subsequently used for qRT-PCR.

### FITC-labeled dextran uptake

After negative selection, enriched PBMCs were incubated with 1mg/mL of FITC-labeled dextran (MW 40000; Sigma–Aldrich) for 1 h at 37°C or 4°C. The cells were washed four times with phosphate-buffered saline and was analyzed by flow cytometry.

### Mixed-lymphocyte culture (MLC) assay

MLC assays were conducted in 96-well round-bottom plates. Carboxyfluorescein succinimidyl ester (CFSE)-labeled CD4^+^ T cells were used as responder cells, and the sorted DCs were used as effectors. CD4^+^ T cells were purified using anti-human CD4 microbeads (Miltenyi Biotec). Purified CD4^+^T cells were CFSE-labeled according to the instructions provided by the manufacturer (Molecular Probes, Carlsbad, CA, USA). The final volume of each well was 200 μL. Responder cells (50,000 cells/well) and effector cells (10,000 cells/well) were incubated for 72 h at 37°C in 5% CO_2_, with or without CpG (1 μM) or phytohemagglutinin (PHA; 1 μM; Sigma–Aldrich) stimulation. T-cell proliferation, indicated by CFSE dilution, was analyzed by flow cytometry.

### DC proliferation assay

DC proliferation was analyzed by flow cytometry. Sorted DCs were labeled by CFSE according to the protocol provided by the manufacturer. CFSE-labeled cells were cultured in RPMI medium supplemented with 10% fetal bovine serum and containing 100 ng/mL recombinant human FLT3-L (R&D Systems) and 100 ng/mL GM-CSF (R&D Systems) for six days. Proliferation, which was indicated by CFSE dilution, was analyzed by flow cytometry.

### Statistical methods

Statistically significant differences were determined using one-way analysis of variance followed by Bonfferoni’s post-hoc comparision tests. A *P*-value of <0.05 was considered to be statistically significant. Statistical analysis was performed using GraphPad Prism 6.0 software (GraphPad Software, Inc., San Diego, CA, USA).

## Results

### CD56^+^ pDC-like cells (pDLCs) are rare in the peripheral blood and morphologically resemble pDCs

**Figure 1:**
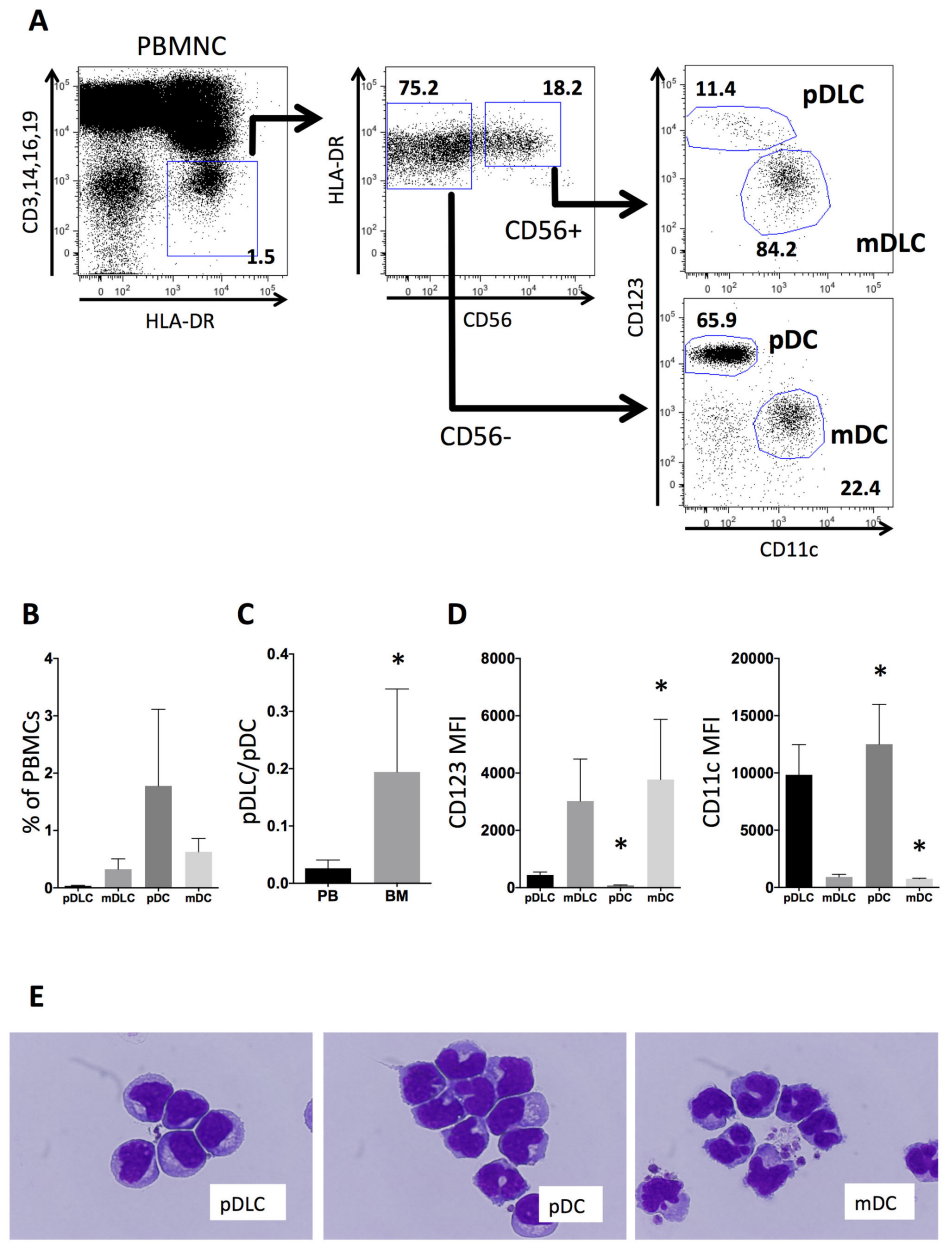
pDLCs are a rare DC population that are morphologically similar to pDCs. (A) PBMNCs were analyzed using flow cytometry. The gated Lin^-^HLA−DR^+^ population (left) was divided according to CD56 expression (middle), and these two subpopulations were analyzed according to CD123 and CD11c expression to define pDLCs, mDLCs, pDCs, and mDCs (right). The results of one representative experiment are presented. (B) The frequency of pDLC, mDLCs, pDCs, and mDCs in PBMCs (n = 5). pDLCs comprised a very small (0.03%) proportion in PBMCs. (C) The pDLC to pDC ratio was compared between peripheral blood (PB) and bone marrow (BM) cells (n=5). *P < 0.05 compared to PB. (D) MFI of CD123 (left panel) and CD11c (right panel) expression was measured on pDLCs, pDCs, and mDCs in PBMCs (n = 5). *P < 0.05 compared to pDLC. (E) Sorted cells were stained with May–Giemsa solution and observed by light microscopy at 1000× magnification.

As displayed in Figure 1A, Lin^−^HLA−DR^+^ cells were sorted into two populations: CD56^+^ and CD56^−^, each of which was further fractionated into two subpopulations according to CD123 and CD11c expression. pDLCs were defined as Lin^−^DR^+^CD56^+^CD123^+^ cells, mDC-like cells (mDLCs) as Lin^−^DR^+^CD56^+^CD123^−^CD11c^+^ cells, pDCs as Lin^−^DR^+^CD56^−^CD123^+^CD11c^−^ cells, and mDCs as Lin^−^DR^+^CD56^−^CD123^−^CD11c^+^ cells.

In PBMCs obtained from healthy volunteers (n = 5), the overall frequency of each population was as follows: pDLC (0.03 ± 0.01%), mDLC (0.27 ± 0.18%), pDC (1.52 ± 1.33%), and mDC (0.66 ± 0.23%, Figure 1B). The pDLC/pDC ratio was higher in the bone marrow than in the peripheral blood (0.129 ± 0.144 vs. 0.02 ± 0.01, n = 5, Figure 1C).

pDLCs exhibited lower mean fluorescence intensities (MFIs) of CD123 and higher MFIs of CD11c compared with those of pDCs (Figure 1D). To examine the morphological characteristics, pDLCs, pDCs, and mDCs were sorted and subsequently stained with May–Giemsa solution. Using light microscopy, pDLCs revealed oval or U-shaped nuclei with condensed chromatin. Furthermore, a perinuclear halo, which is a specific feature of pDCs, was clearly observed in the cytoplasm (Figure 1E).

### pDLCs have an immunophenotype that is similar to pDCs, but share some features with mDCs

**Figure 2:**
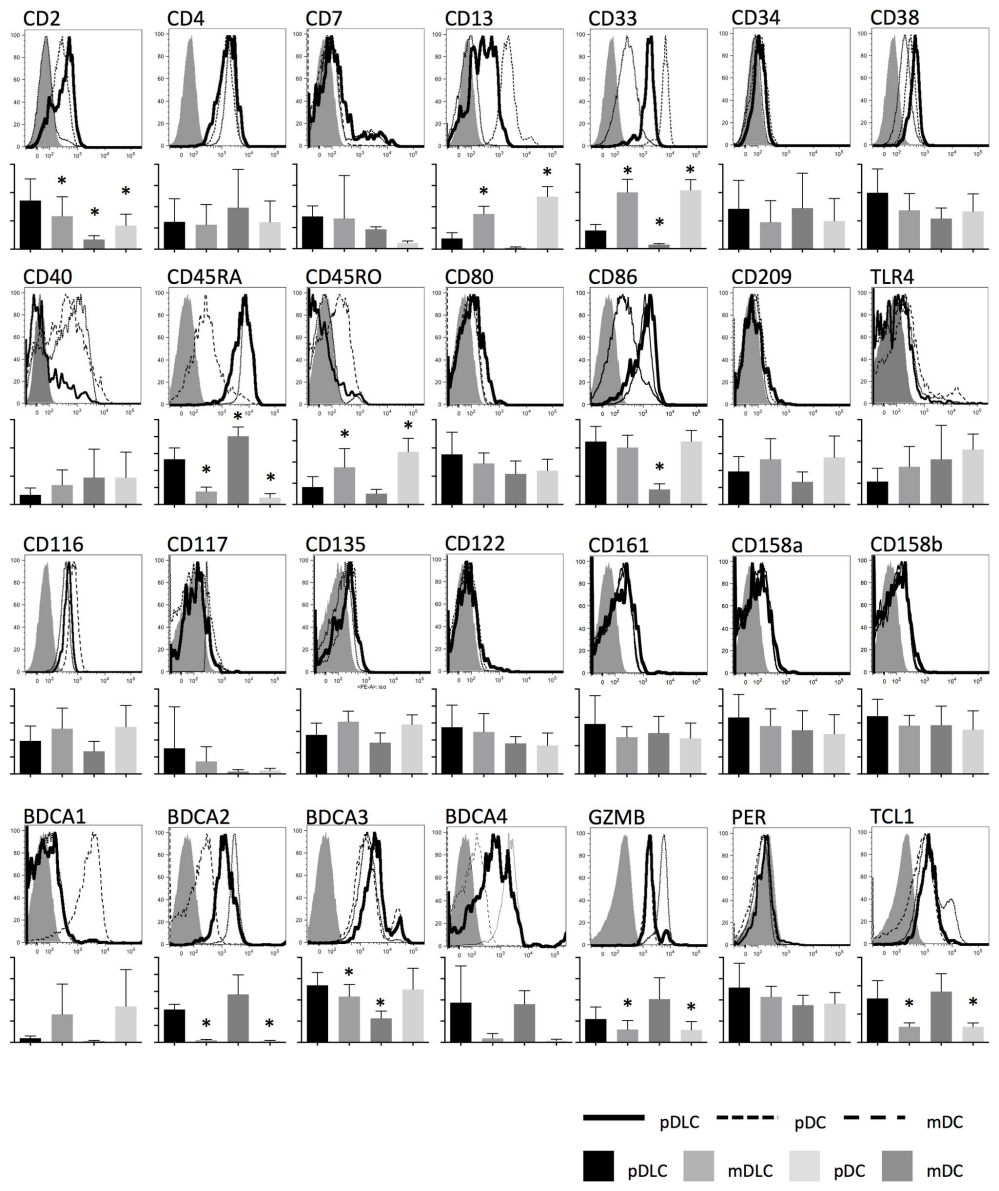
Immunophenotypic characteristics of pDLCs. Expression of cell-surface and intracellular markers in pDLCs, mDLCs, pDCs, and mDCs were measured by flow cytometry. Upper graph showed the results of one representative experiment. pDLCs (solid line), pDCs (dotted line), mDCs (dashed line) and isotype control (filled histogram). Lower graph showed mean fluorescence intensity of pDLC, mDLC, pDC and mDC (n=5). * P < 0.05 compared to pDLC.

Immunophenotypical characteristics of pDLCs, mDLCs, pDCs, and mDCs were analyzed using flow cytometry (Figure 2). Although Lin^−^CD56^+^ cells usually represent NK cells, the absence of several NK cell-specific antigens (e.g., CD122, CD161, CD158a, and CD158b) confirmed that pDLCs were not NK cells. Both pDLCs and pDCs were positive for CD4, CD38, CD45RA, CD116, BDCA2, and BDCA4. In addition, pDLCs were positive for CD2, CD13, and CD33, antigens which are generally expressed in mDCs, but not pDCs.

### pDLCs express TLRs at an intermediate level between pDC and mDC

**Figure 3:**
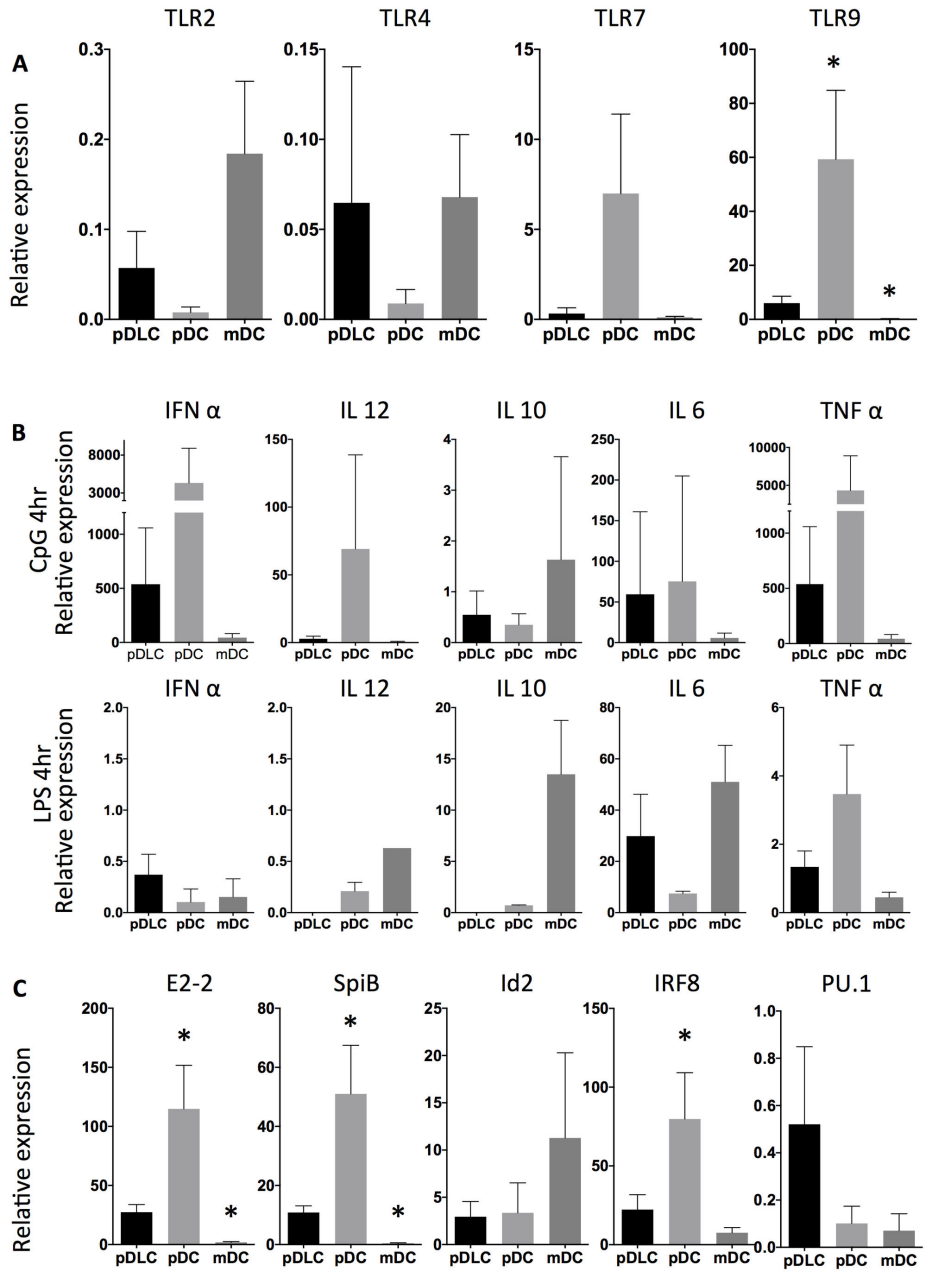
TLR, cytokine, and transcription factor expression levels in pDLCs are distinct from pDCs and mDCs. qRT-PCR analyses were performed using cDNA from pDLCs, pDCs, and mDCs. Results were standardized using PBMC cDNA (n=5). * P < 0.05 compared to pDLC. (A) TLR expression analysis revealed that mDCs expressed TLR2 and TLR4, whereas pDCs expressed TLR7 and TLR9. (B) Cytokine production was analyzed after CpG or LPS stimulation. (C) Transcripts of E2-2, Id2, Irf8, PU.1, and SpiB were analyzed in pDLCs, pDCs, and mDCs.

TLRs are critical molecules for DC activation. This study investigated the TLR expression profile of three DC subpopulations (pDLC, pDC, and mDC) by qRT-PCR (Figure 3A). TLRs were expressed in pDLCs at an intermediate level between that of pDCs and mDCs in pDLC. In case of TLR4, although we could detect mRNA at least but could not detect the protein with flow cytometry (Figure 3A and Figure 2).

### pDLC cytokine production patterns are distinct from pDC or mDC after TLR stimulation

Cytokine production is critical role of DCs. Especially pDCs are robust IFN-α-producing cells. Cytokine production among pDLC, pDC, and mDC after CpG or LPS stimulation were evaluated by qRT-PCR (Figure 3B). Levels of IFN-α and TNF-α expression after CpG stimulation were abundant in pDCs but low in pDLCs and mDCs. Furthermore, IL-10 and IL-12 production after LPS stimulation were high in mDCs but low in pDLCs and pDCs. These results were consistent with the low expression of the CpG receptor TLR9 in pDLCs. In addition, IL-10 and IL-12 production in response to LPS was lower in pDLCs than in mDCs. Those were caused by low expression of TLR4 with flow cytometry in pDLCs.

### pDC-specific transcription factors are expressed at an intermediate level in pDLC

Recent research has characterized the role of transcription factors in DC differentiation. The expression of various transcription factors such as E2-2, Irf8, Id2, PU.1, and SpiB was examined in various cell types by qRT-PCR (Figure 3C). In pDLCs, expression of transcription factors, including Ets family, Spi-B, E-box protein, E2-2, and Irf8 was lower than that in pDCs but higher than that in mDCs (Figure 3C). PU.1 expression, a gene involved in the differentiation of murine DCs precursors, was higher in pDLCs than in pDCs (Figure 3C).

### pDLCs have lower endocytic activity than mDCs and induce less proliferation of CD4^+^T cells than that in pDCs

**Figure 4:**
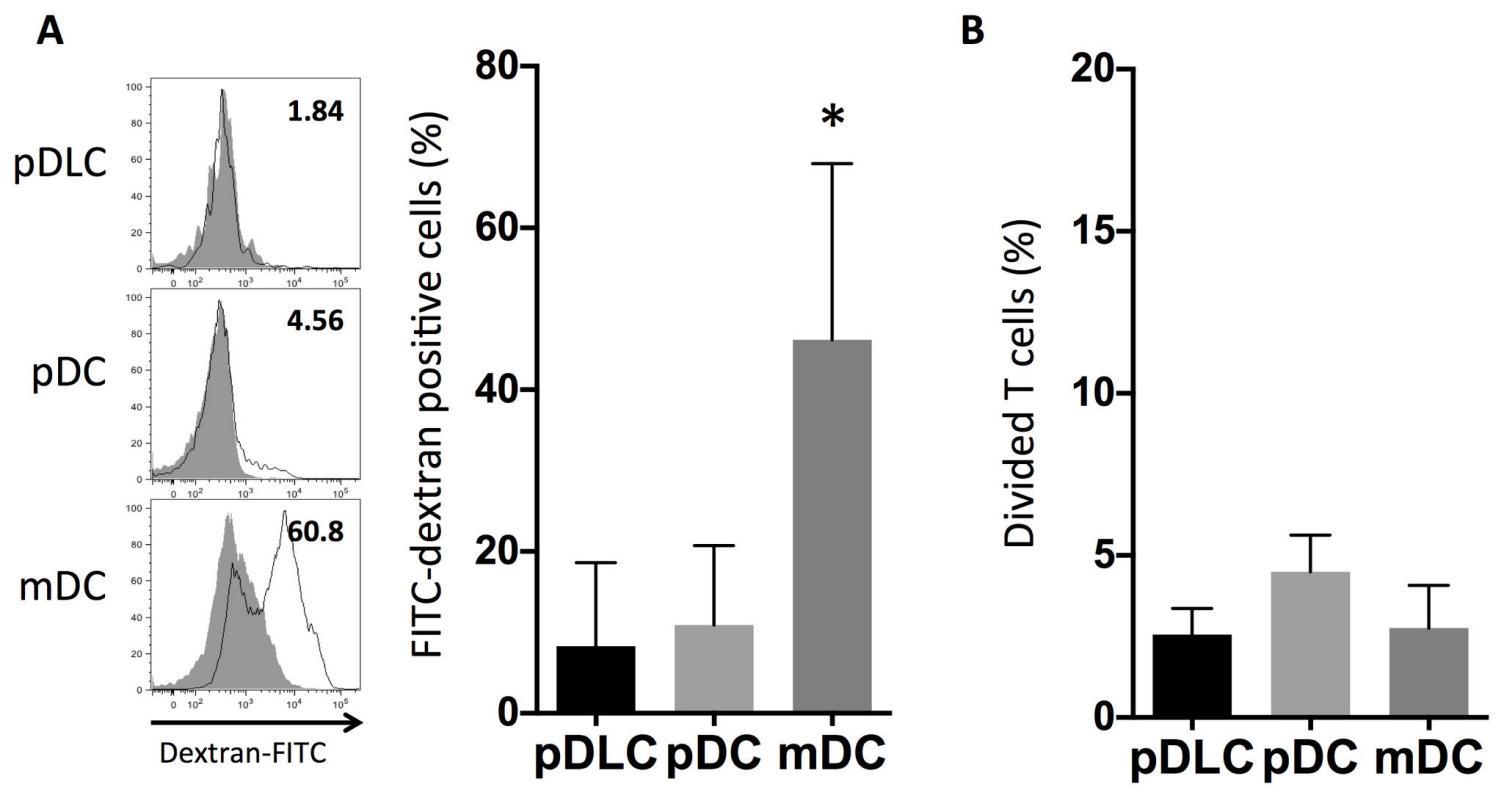
pDLCs exhibit lower endocytic activity than that in mDCs, and also induce less proliferation of CD4^+^T cells than that in pDCs. * P < 0.05 compared to pDLC. (A) PBMCs were incubated with FITC–dextran and analyzed by flow cytometry. The open histograms represent 37°C- and the filled histograms represent 4°C-cultured cells. Results of one representative experiment are presented (left). The average frequencies of FITC–dextran positive cells are indicated (n = 5, right). (B) Sorted DCs were co-cultured for 72 h with CFSE-labeled CD4^+^ T cells under CpG stimulation (1µM). The average frequencies of divided CD4 positive cells are indicated (n = 5).

Endocytosis is a functional feature of mDCs. PBMCs were incubated at 37°C or 4°C as a control, with FITC-labeled dextran for 1 h, and the frequency of FITC-labeled dextran-positive cells was assessed using flow cytometry (Figure 4A). Compared with 4°C controls, mDCs revealed higher uptake levels of FITC-labeled dextran than that in both pDLCs and pDCs.

To determine whether pDLCs could stimulate T-cell proliferation, sorted pDLCs, pDCs, or mDCs were stimulated with CpG and cocultured with allogeneic T cells (Figure 4B). Furthermore, compared with pDCs, both pDLCs and mDCs induced fewer proliferating cells. These data indicated that compared with pDCs, pDLCs had reduced capacity of prime T-cell proliferation after CpG stimulation.

### pDLC express mDC-specific antigens when stimulated with Flt3-Ligand and GM-CSF

**Figure 5:**
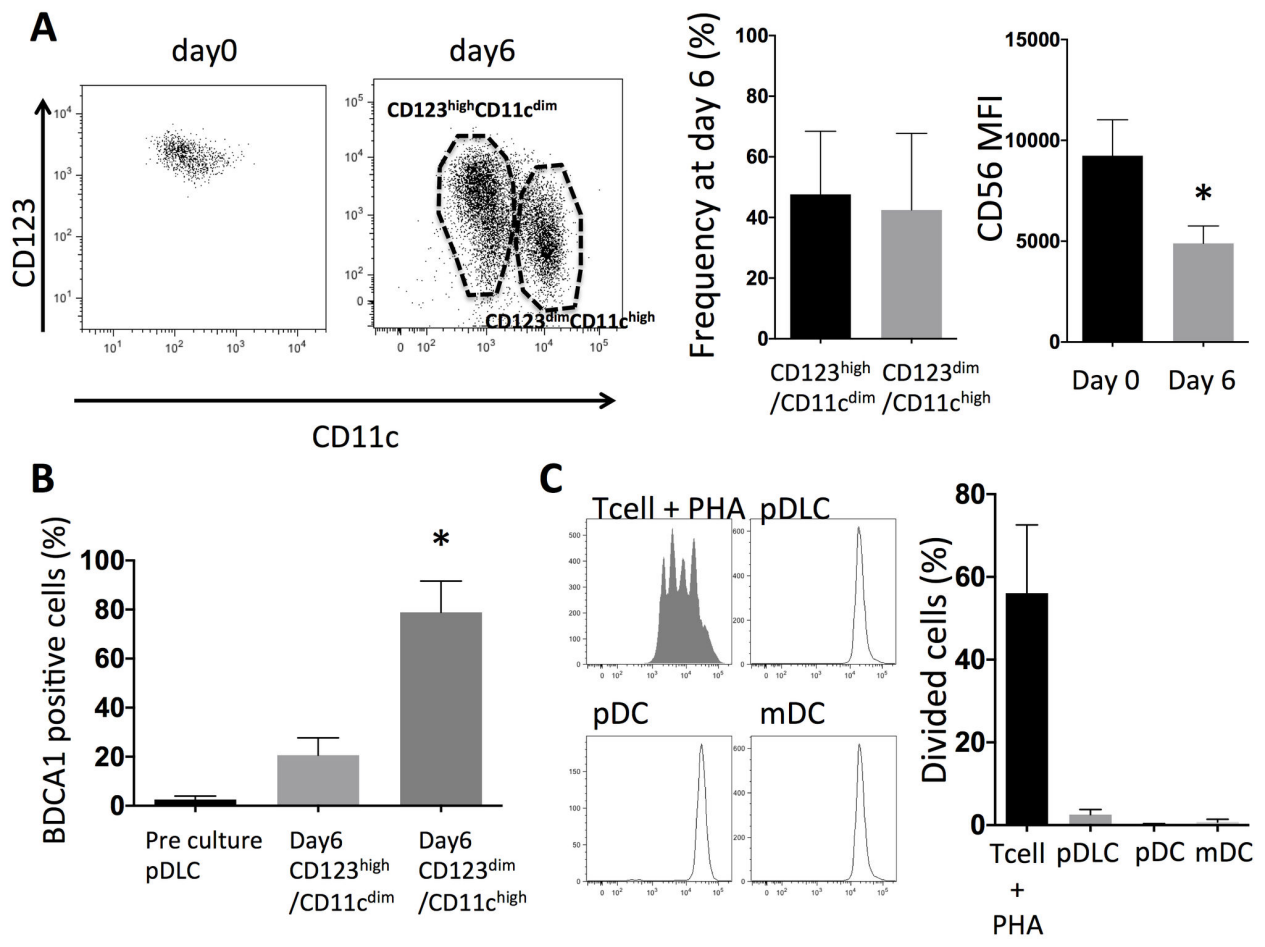
pDLCs expressed mDC specific antigens. pDLCs were cultured with Flt3-Ligand GM-CSF for six days. (A) CD123, CD11c and CD56 expressions were analyzed at day 0 and 6. CD123^high^CD11c^dim^ and CD123^dim^CD11c^high^ populations were observed at day 6. Results of one representative experiment are presented (left). The average frequencies of CD123^high^CD11c^dim^ and CD123^dim^CD11c^high^ populations are indicated (n = 5, middle). The average MFI of CD56 at day 0 and 6 are indicated (n = 5, right). * P < 0.05 compared to day0. (B) BDCA1 expression was compared by flow cytometry. pDLCs did not express BDCA1 at day 0, but they did express BDCA1 at day 6 especially CD123^dim^CD11c^high^ population. * P < 0.05 compared to day0 (n = 5). (C) Sorted cells were CFSE-labeled and incubated for six days with Flt3-L and GM-CSF. The filled histograms represent PHA-stimulated CFSE-labeled CD4^+^ T cells that were included as a control for cell division (left). The average frequencies of divided cells are indicated (n = 5, right).

pDLCs were cultured with Flt3-L (100 ng/mL) and GM-CSF (100 ng/mL) for 6 days. A CD123^dim^CD11c^high^ population and a CD123^high^CD11c^dim^ population appeared at day 6 (Figure 5A). CD56 expression was decreased at day 6 compared to that of pDLC at day0. BDCA1 expression in pDLCs was absent at day 0 but abundant at day 6 especially in CD123^dim^CD11c^high^ population (Figure 5B). On the other hand CD123^dim^CD11c^high^ population was not observed when pDCs were cultured with Flt3-L and GM-CSF for 6 days. In order to rule out the possibility that these BDCA1-expressing cells represented contaminating cells that had proliferated in the culture, the proliferation activity of purified pDLC cells was analyzed. CFSE-labeled cells were analyzed by flow cytometry after being cultured for six days with Flt3-Land GM-CSF; however, no CFSE dilution was observed. These data indicated that the expression of cell-surface markers by purified pDLCs changed after *in vitro* culture. Moreover, these populations were phenotypically similar to pDCs and mDCs.

## Discussion

This study investigated the immunophenotype, function, and transcriptional factor expression of pDLCs and compared them with those of pDC, the putative normal counterpart of BPDCN. Initially, we had to exclude the possibility that this population was composed of a mixture of several cell lineages (e.g., pDC and mDC). Our finding that pDLCs had one large peak for many antigens by flow cytometry, such as BDCA2, CD33, and CD45RA, indicated that they represented a distinct population.

pDLCs expressed many antigens that were specific to pDCs, including BDCA2, BDCA4, CD40, CD45RA, and CD86, but they also expressed antigens that were atypical for pDCs and had been frequently reported for BPDCN, such as CD2, CD13, and CD33. These findings suggest that pDLCs are a distinct type of DC, which are more similar to pDCs than mDLCs and mDCs, particularly regarding BDCA antigen expression[9]. Furthermore, the complex immunophenotype resembles the heterogeneous immunophenotype of BPDCN. Although one study suggested that pDC in humans treated with Flt3-L changed in immunophenotype (e.g., CD56 expression), this population exhibited lower expressions of CD45RA and CD56[19]. pDC can express CD56 in response to stimuli that promote maturation of immature DCs other than Flt3-L that promote differentiation from stem cell to DCs[18,20,21]. However, expression patterns of CD56 and other antigens in activated pDC are also different from those of pDLC in steady-state and for BPDCN.

The expression profile of transcriptional factors in pDLC was distinct from those of pDC and mDC. Furthermore, the expression levels of transcriptional factors, which are specific for pDC differentiation (E2-2 and SpiB) in pDLCs, were intermediate, between the levels in pDCs and mDCs. Id2 blocks pDC differentiation from CDP, and expression of Id2 in pDLC was intermediate as well, between the levels in pDC and mDC[22]. Intriguingly, PU.1 expression, which is directly regulated by Flt3 and is required for GM-CSF-induced DC differentiation in mice, was higher in pDLC than in both pDC and mDC[23]. Considering that these transcriptional factors directly regulate the expression of TLRs[24,25], low expression levels of E2-2 and SpiB suggest possible association with relatively low expression levels of TLRs and other functional molecules in pDLCs. Circulating pDCs mediate rapid and robust secretion of IFN-α following bacterial or virus infection through TLR7 and TLR9 by sensing either non-methylated DNA or RNA. Thus, low expression levels of TLR7 and TLR9 in pDLC accounts for the low IFN-α and TNF-α response after CpG stimulation. LPS stimulation induces IL-10 production in mDC but not in pDLC which express TLR4 at mRNA level but not at protein level. In fact, our data suggested that pDLC also have weak phagocytic function and T cell priming capacity[26,27]. These data indicate that pDLCs are not functionally equivalent to pDCs or BPDCNs, probably due to their distinct transcriptional factor expression profile.

 Transcription factor expression pattern in pDLCs might be similar to that seen in dendritic cell precursors of mice. Moreover dendritic precursors appear to differentiate in the bone marrow[13], and our study revealed that pDLCs reside in relatively higher numbers in the bone marrow. NCAM, corresponding to CD56 in humans, marks immature mouse DCs[15]. On the basis of these data, we speculate that pDLCs could represent an immature stage of developing DCs. Finally, this study revealed that the cytokine combination of Flt3-L and GM-CSF, which can induce CD34^+^ cell differentiation of mDCs[28], could decrease CD56 expression and induce BDCA1 and CD11c expressions. Phenotypically CD123^high^CD11c^dim^ is similar to pDCs and CD123^dim^CD11c^high^ is also similar to mDCs. We observed that our cytokine combination was quite different from stimuli, which induce phenotypic and functional changes from mouse pDC to mDC at mature stage[29]. On the other hand BPDCN cell line, PMDC05, was reported to be consisted of several phenotypically different populations, and one of these populations, CD123^+^BDCA1^-^, changed its phenotype from BDCA1 negative to positive after GM-CSF stimulation[30]. This suggests that BPDCN contains potential, maybe immature, population that gives rise to mDC at least in phenotype. In our study, pDCs cultured with Flt3-L and GM-CSF did not show mDC differentiation even in their phenotype. Considering PMDC05 and pDLC phenotypical change and transcriptional factors expression profile, pDLCs could be another candidate of normal counterpart of BPDCN. Further studies will be required to identify the differentiation stage represented by pDLCs and the relation between BPDCN and pDLC.
